# Dermoscopic Monitoring of Therapeutic Regression in Actinic Lichen Planus: A Practical Illustration of Inflammoscopy in Clinical Care

**DOI:** 10.1155/crdm/5877562

**Published:** 2026-10-21

**Authors:** NagaNitya Vangala, Adarshlata Singh, Bhushan Madke, Varun H.

**Affiliations:** ^1^ Department of Dermatology, Venereology & Leprosy Jawaharlal Nehru Medical College Datta Meghe Institute of Higher Education and Research Deemed to Be University Wardha Maharashtra India dmimsu.edu.in

**Keywords:** actinic lichen planus, dermoscopy, inflammoscopy, lichen planus, therapeutic monitoring

## Abstract

Dermoscopic monitoring has emerged as a valuable noninvasive adjunct for evaluating inflammatory dermatoses by providing objective assessment of disease activity and therapeutic response. Its role in actinic lichen planus (ALP), however, remains incompletely characterized. We report a biopsy‐confirmed case of ALP in a middle‐aged man presenting with asymptomatic hyperpigmented plaques over photoexposed areas of the face. Baseline polarized dermoscopy demonstrated annular Wickham’s striae, a central gray structureless area, gray–brown dots, fine white scales, and diffuse brown pigmentation. Histopathological examination revealed compact orthokeratosis, wedge‐shaped hypergranulosis, basal cell vacuolar degeneration, pigment incontinence, and a dense band–like lichenoid lymphocytic infiltrate, confirming the diagnosis. The patient received combined therapy comprising oral hydroxychloroquine, topical corticosteroid therapy, broad‐spectrum photoprotection, and two sessions of fractional CO_2_ laser performed at 4‐week intervals. At the 8‐week follow‐up, clinical improvement was accompanied by attenuation of inflammatory dermoscopic structures with persistence of residual pigmentary features, reflecting regression of active interface dermatitis despite ongoing postinflammatory pigmentation. This case highlights the clinicodermoscopic and histopathological correlation in ALP and illustrates the practical utility of dermoscopic monitoring (inflammoscopy) as a noninvasive adjunct for assessing therapeutic response in routine clinical practice.

## 1. Introduction

Actinic lichen planus (ALP), also known as lichen planus actinicus or lichen planus subtropicus, is a rare photodistributed variant of lichen planus that predominantly affects individuals with darker skin phototypes residing in tropical and subtropical regions. It typically presents during the spring and summer months as asymptomatic or mildly pruritic hyperpigmented, annular, or dyschromic plaques involving chronically sun‐exposed areas, particularly the forehead, temples, cheeks, dorsum of the hands, and forearms. Although the exact pathogenesis remains incompletely understood, ultraviolet radiation is believed to act as an important environmental trigger in genetically susceptible individuals. Unlike classical lichen planus, persistent postinflammatory hyperpigmentation frequently dominates the clinical picture and may continue long after inflammatory activity has subsided, making assessment of disease activity based solely on clinical examination difficult [1–3].

Dermoscopy has become an important noninvasive adjunct in the evaluation of inflammatory dermatoses by enabling in vivo visualization of structures that correlate with histopathological alterations. In lichen planus, dermoscopic examination typically demonstrates Wickham’s striae, vascular structures, scaling, and pigmentary changes, with the relative prominence of these features varying according to disease activity and stage [4, 5]. Previous studies have described conspicuous Wickham’s striae and inflammatory structures in active lesions, with attenuation of these features and persistence of gray–brown pigmentation during regression [5, 6]. Although the dermoscopic features of classical lichen planus have been extensively investigated, published literature specifically describing ALP, particularly during treatment, remains limited [6, 7].

The expanding application of dermoscopy in inflammatory skin diseases has led to the concept of inflammoscopy, whereby dermoscopy is used not only for diagnosis but also for longitudinal assessment of inflammatory activity and treatment response. By allowing serial comparison of disease‐associated dermoscopic structures, this approach may provide a noninvasive adjunct for monitoring therapeutic response. This may be particularly useful in ALP, where persistent pigmentation can obscure clinical improvement and complicate assessment based on naked‐eye examination alone [8, 9].

The present report describes a biopsy‐confirmed case of ALP in which baseline and follow‐up polarized dermoscopic examinations demonstrated regression of inflammatory dermoscopic structures following combined therapy, despite persistent residual pigmentation. Through clinicodermoscopic and histopathological correlation, this case highlights the practical role of dermoscopic monitoring as an adjunctive tool for assessing therapeutic response and illustrates the clinical utility of inflammoscopy in routine dermatological practice.

## 2. Case Report

A middle‐aged man presented to our dermatology outpatient department with an 8–12‐month history of progressively increasing asymptomatic hyperpigmented lesions involving the forehead and other photoexposed areas of the face. There was no history of preceding erythema, photosensitivity, oral or genital lesions, scalp involvement, pruritus, or similar lesions elsewhere on the body. His past medical, drug, and family histories were unremarkable, and systemic examination did not reveal any abnormalities.

Cutaneous examination revealed multiple well‐defined hyperpigmented to violaceous‐brown plaques over the forehead, with additional hyperpigmented patches involving other sun‐exposed facial areas (Figure 1A). Mucosal, nail, and scalp examinations were normal. Based on the clinical morphology and photodistribution, the differential diagnoses included ALP, lichen planus pigmentosus, melasma, discoid lupus erythematosus, and facial acanthosis nigricans.

**FIGURE 1:**
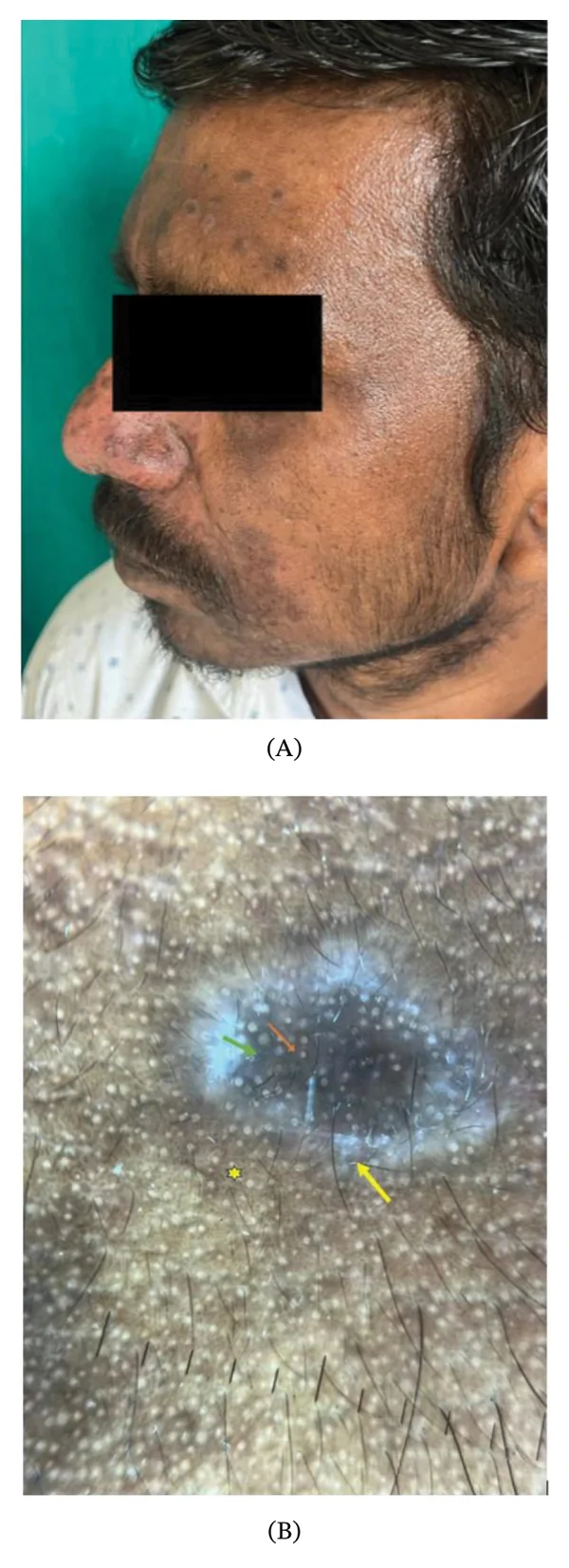
(A) Clinical photograph at presentation showing multiple well‐defined hyperpigmented to violaceous‐brown plaques over the forehead with additional hyperpigmented patches involving other photoexposed areas of the face. The yellow circle indicates the representative lesion selected for baseline dermoscopic examination and pretreatment punch biopsy. (B) Baseline polarized dermoscopy (DermLite DL5, × 10) of the representative lesion demonstrating annular Wickham’s striae (blue arrows), a central gray structureless area (green arrow), white rosettes (red arrow), fine white scales (yellow arrow), gray–brown dots corresponding to dermal melanophages (orange arrow), and diffuse background brown pigmentation (yellow star).

To further characterize the representative forehead lesion, baseline polarized dermoscopic examination (DermLite DL5, × 10) was performed. Dermoscopy demonstrated annular Wickham’s striae, a central gray structureless area, gray–brown dots, fine white scales, and diffuse brown pigmentation (Figure 1B), findings that favored the diagnosis of ALP.

A pretreatment 4‐mm punch biopsy was subsequently obtained from the same representative forehead lesion examined dermoscopically. Histopathological examination demonstrated compact orthokeratosis, wedge‐shaped hypergranulosis, irregular acanthosis, basal cell vacuolar degeneration, dense band–like lichenoid lymphocytic infiltrate along the dermoepidermal junction, and pigment incontinence with dermal melanophages, confirming the diagnosis of ALP (Figure 2A and B).

**FIGURE 2:**
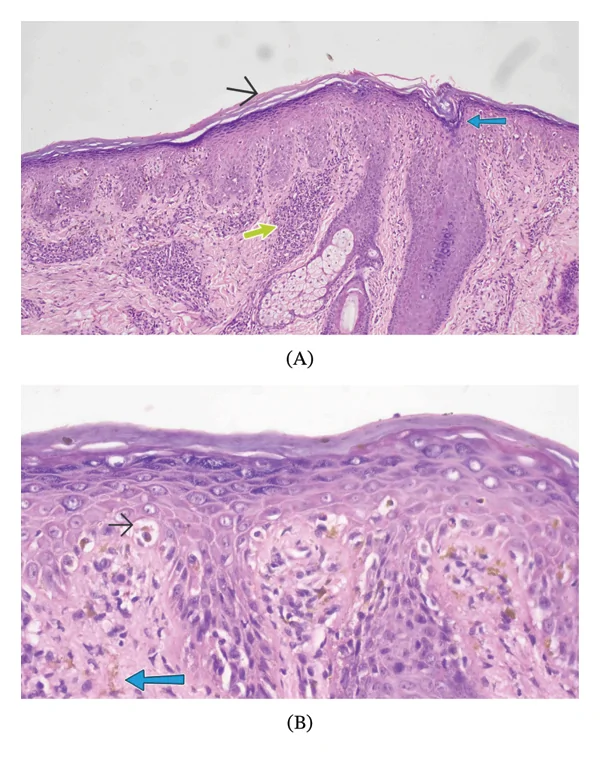
(A) Pretreatment punch biopsy from the representative forehead lesion showing compact orthokeratosis (black arrow), wedge‐shaped hypergranulosis (blue arrow), dense band‐like lichenoid lymphocytic infiltrate along the dermoepidermal junction (green arrow), and focal pigment incontinence in the superficial dermis (hematoxylin and eosin, × 100). (B) Higher‐power photomicrograph demonstrating basal cell vacuolar degeneration (black arrow), dense lichenoid lymphocytic infiltrate at the dermoepidermal junction, and pigment incontinence with dermal melanophages (blue arrows) (hematoxylin and eosin, × 400).

The patient was treated with oral hydroxychloroquine (200 mg once daily), topical mometasone furoate 0.1% cream, strict photoprotection with broad‐spectrum sunscreen, and fractional CO_2_ laser therapy, with two treatment sessions performed at 4‐week intervals before follow‐up evaluation.

At the 8‐week follow‐up, the forehead plaques demonstrated appreciable flattening with a marked reduction in pigmentation (Figure 3A). Repeat polarized dermoscopic examination of the same representative lesion revealed attenuation of inflammatory dermoscopic structures, with persistence of residual pigmentary features including brown pigmentation, a central gray structureless area, and fine white scales (Figure 3B).

**FIGURE 3:**
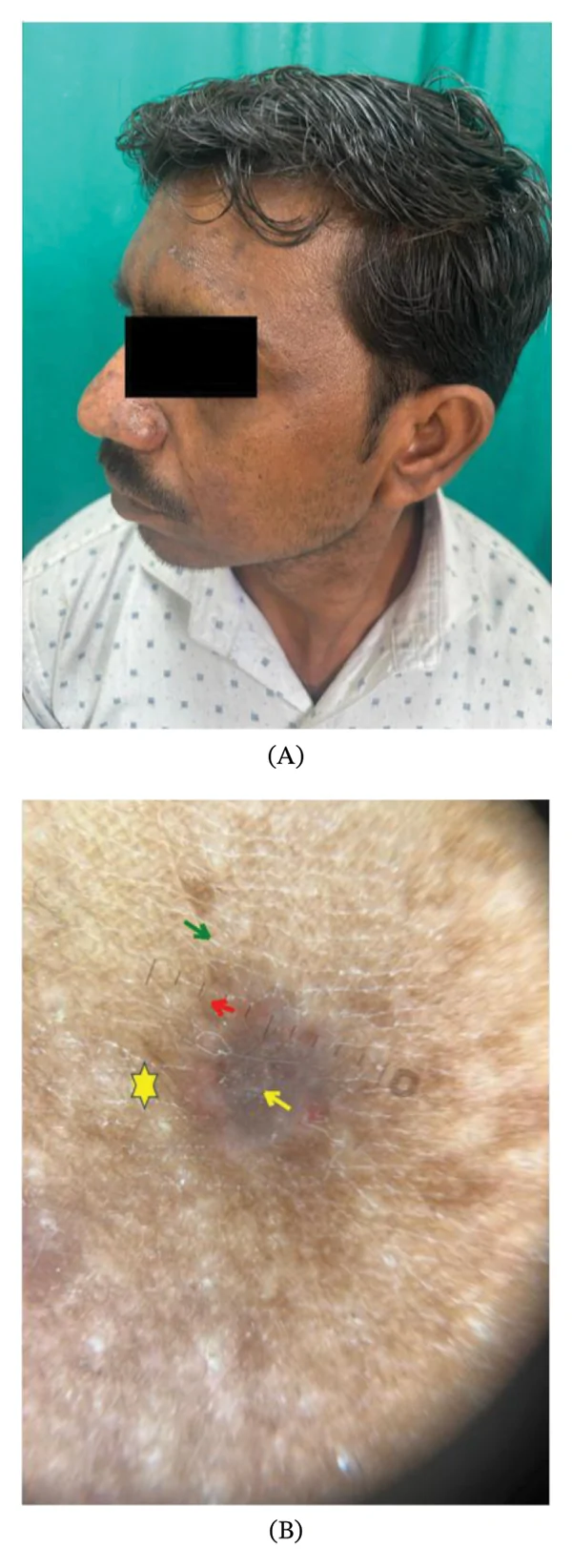
(A) Clinical photograph obtained after 8 weeks of combined therapy demonstrating flattening and lightening of the forehead plaques with reduction in overall pigmentation, consistent with clinical improvement. The yellow circle indicates the same representative lesion that underwent baseline and follow‐up dermoscopic examination. (B) Follow‐up polarized dermoscopy (DermLite DL5, × 10) of the same representative lesion after 8 weeks of combined therapy demonstrating fine white scale (green arrow), brown pigmentation (red arrow), central gray structureless area (yellow arrow), and superficial white scales (yellow star).

## 3. Discussion

ALP is an uncommon photodistributed variant of lichen planus in which persistent postinflammatory pigmentation frequently obscures clinical assessment of disease activity. In the present case, biopsy‐confirmed ALP demonstrated marked clinical improvement following combined therapy; however, residual pigmentation persisted despite attenuation of inflammatory changes. Baseline and follow‐up polarized dermoscopic examination of the same representative lesion provided additional objective evidence of change in inflammatory dermoscopic structures, thereby complementing routine clinical examination. This observation highlights the potential role of dermoscopic monitoring as a practical adjunct for assessing therapeutic response in ALP, where clinical improvement may be difficult to appreciate because of persistent pigmentary sequelae [1–3, 7].

The dermoscopic findings observed in our patient closely paralleled the underlying histopathological alterations. Baseline dermoscopy demonstrated annular Wickham’s striae, gray–brown dots, fine white scales, a central gray structureless area, and diffuse brown pigmentation. Histopathological examination revealed compact orthokeratosis, wedge‐shaped hypergranulosis, basal cell vacuolar degeneration, dense lichenoid lymphocytic infiltration along the dermoepidermal junction, and pigment incontinence with dermal melanophages. Wickham’s striae are well‐recognized dermoscopic correlates of compact orthokeratosis and wedge‐shaped hypergranulosis, whereas gray–brown dots and structureless gray areas correspond to dermal melanophages resulting from pigment incontinence secondary to interface damage. Fine white scales reflect surface hyperkeratosis, while diffuse brown pigmentation represents epidermal hypermelanosis with variable dermal melanin deposition. Similar clinicopathological correlations have been described by Friedman et al., Güngör et al., and Singh et al., supporting the clinicodermoscopic correlation and diagnostic utility of dermoscopy in lichen planus and its actinic variant [4–7].

At the 8‐week follow‐up, after completion of two fractional CO_2_ laser sessions in combination with oral hydroxychloroquine, topical corticosteroid therapy, and photoprotection, repeat dermoscopy demonstrated attenuation of inflammatory dermoscopic structures with persistence of residual pigmentary features. Although the forehead plaques showed appreciable flattening and reduction in pigmentation clinically, residual gray pigmentation remained evident dermoscopically. These findings are most likely consistent with persistence of dermal melanophages despite resolution of active interface inflammation. Similar observations have been reported in studies evaluating active and regressive lichen planus, where inflammatory dermoscopic structures diminish during treatment, while pigmentary features may persist because of delayed clearance of dermal melanin [5, 6].

The expanding application of dermoscopy in inflammatory dermatoses has led to the concept of inflammoscopy, in which dermoscopic examination is used not only for diagnosis but also for longitudinal assessment of inflammatory activity and therapeutic response. By enabling serial comparison of disease‐associated dermoscopic structures, inflammoscopy provides a noninvasive approach to monitoring disease evolution. In disorders such as lichen planus and other inflammatory dermatoses, dermoscopic changes may complement clinical assessment and, in some settings, parallel histopathological or treatment‐associated changes. In ALP, where persistent pigmentation often obscures therapeutic improvement, dermoscopic monitoring may help clinicians distinguish residual pigmentary change from persistent inflammatory activity [8, 9].

Although dermoscopic features of ALP have been previously described, reports documenting dermoscopic evolution during treatment remain limited. The educational value of the present case lies not in demonstrating a novel dermoscopic feature, but in illustrating the clinicodermoscopic and histopathological correlation of therapeutic regression in biopsy‐confirmed ALP. Furthermore, documenting the same representative lesion before treatment and after 8 weeks of follow‐up provides a practical example of how dermoscopy can complement routine clinical examination in evaluating treatment response in pigmentary variants of lichen planus [4–7, 9].

This report highlights the practical value of dermoscopic monitoring as a noninvasive adjunct for assessing therapeutic response in biopsy‐confirmed ALP. By demonstrating clinicodermoscopic and histopathological correlation before and after treatment, it illustrates how serial dermoscopy may assist in distinguishing persistent inflammatory activity from residual postinflammatory pigmentation, particularly when clinical assessment alone may be insufficient. Although limited by the experience of a single patient and evaluation at two time points, this case supports the potential use of dermoscopic monitoring in routine follow‐up of pigmentary variants of lichen planus and underscores the need for larger prospective studies to validate standardized dermoscopic markers of disease activity and therapeutic response [8, 9].

## Author Contributions

Naga Nitya Vangala: conceptualization, patient management, dermoscopic evaluation, data collection, literature review, manuscript drafting, figure preparation, and revision of the manuscript. Adarshlata Singh: clinical supervision, patient management, critical revision of the manuscript, and final approval of the version to be published. Bhushan Madke: histopathological interpretation, clinical supervision, critical intellectual review of the manuscript, and final approval of the version to be published. Varun H.: data collection, literature review, manuscript editing, and final approval of the version to be published.

## Funding

The authors received no specific funding for this work.

## Disclosure

All authors read and approved the final manuscript.

## Ethics Statement

Institutional ethics committee approval was not required for this case report in accordance with institutional policy.

## Consent

Written informed consent was obtained from the patient for publication of this case report and the accompanying clinical and histopathological images. A copy of the written consent is available for review by the Editor‐in‐Chief upon reasonable request.

## Conflicts of Interest

The authors declare no conflicts of interest.

## Supporting Information

Additional supporting information can be found online in the Supporting Information section.

## Supporting information


**Supporting Information** CARE checklist.

## Data Availability

The data supporting the findings of this case report are available from the corresponding author upon reasonable request. All data have been anonymized to protect patient confidentiality.
